# Threats to patient safety in primary care reported by older people with multimorbidity: baseline findings from a longitudinal qualitative study and implications for intervention

**DOI:** 10.1186/s12913-017-2727-9

**Published:** 2017-11-21

**Authors:** Rebecca Hays, Gavin Daker-White, Aneez Esmail, Wendy Barlow, Brian Minor, Benjamin Brown, Thomas Blakeman, Caroline Sanders, Peter Bower

**Affiliations:** 1 NIHR Greater Manchester Primary Care Patient Safety Translational Research Centre (Greater Manchester PSTRC), Division of Population Health, Health Services Research and Primary Care, School of Health Sciences, Faculty of Biology, Medicine and Health, University of Manchester, Manchester Academic Health Science Centre, Manchester, UK; 2 Centre for Health Informatics, School of Health Sciences, Faculty of Biology, Medicine and Health, University of Manchester, Manchester Academic Health Science Centre, Manchester, UK; 3 NIHR Collaboration in Applied Health Research and Care Greater Manchester, Division of Population Health, Health Services Research and Primary Care, School of Health Sciences, Faculty of Biology, Medicine and Health, University of Manchester, Manchester Academic Health Science Centre, Manchester, UK; 4 NIHR School for Primary Care Research, Division of Population Health, Health Services Research and Primary Care, School of Health Sciences, Faculty of Biology, Medicine and Health, University of Manchester, Manchester Academic Health Science Centre, Manchester, UK

**Keywords:** Patient safety, Primary care, Multimorbidity, Older people, Qualitative study

## Abstract

**Background:**

In primary care, older patients with multimorbidity (two or more long-term conditions) are especially likely to experience patient safety incidents. Risks to safety in this setting arise as a result of patient, staff and system factors; particularly where these interact or fail to do so.

Recent research and policy highlight the important contribution patients can make to improving safety. Older patients with multimorbidity may have the most to gain from increasing their involvement but before interventions can be developed to support them to improve their patient safety, more needs to be known about how this is threatened and how patients respond to perceived threats.

We sought to identify and describe threats to patient safety in primary care among older people with multimorbidity, to provide a better understanding of how these are experienced and to inform the development of interventions to reduce risks to patient safety.

**Methods:**

Twenty-six older people, aged 65 or over, with multimorbidity were recruited to a longitudinal qualitative study. At baseline, data on their health and healthcare were collected through semi-structured interviews. Data were analysed thematically, using a framework developed from a previous synthesis of qualitative studies of patient safety in primary care.

**Results:**

Threats to patient safety were organised into six themes, across three domains of health and care. These encompassed all aspects of the patient journey, from access to everyday management. Across the journey, many issues arose due to poor communication, and uncoordinated care created extra burdens for patients and healthcare staff. Patients’ sense of safety and trust in their care providers were especially threatened when they felt their needs were ignored, or when they perceived responses from staff as inappropriate or insensitive.

**Conclusions:**

For older patients with multimorbidity, patient safety is intrinsically linked to the challenges people face when managing health conditions, navigating the healthcare system, and negotiating care. We consider the implications of this for the development of interventions to reduce threats to patient safety. Potential patient-centred mechanisms include providing patients with more realistic expectations for primary care, and supporting them to communicate their needs and concerns more effectively.

**Electronic supplementary material:**

The online version of this article (doi: 10.1186/s12913-017-2727-9) contains supplementary material, which is available to authorized users.

## Background

Patient safety is defined as “the avoidance, prevention and amelioration of adverse outcomes or injuries stemming from the process of healthcare” [1], while harm arising from a patient safety incident is defined as “impairment of structure or function of the body and/or any deleterious effect arising there from, including disease, injury, suffering, disability and death, and may be physical, social or psychological” [2].

A significant number of patient safety incidents arise in primary care (defined here as general or family practice). Record review studies have identified a median of 2–3 incidents per 100 consultations [3]. A recent review of patient safety incident reports from general practice found those concerning assessment or diagnosis were related to the highest proportion of patient harm [4]. The authors identified four drivers of patient safety incidents: communication in referral and discharge; professional decision-making; symptom presentation that delayed cancer diagnosis; and failure to identify patient deterioration and subsequent management delays.

Record review studies show the number and type of incidents reported in primary care but there is also need to develop a broader understanding of how and when safety can be threatened. That is, potential risks to patient safety or precursors to patient safety incidents. A recent review of qualitative studies of patient safety in primary care considered observations of practice alongside stakeholder perceptions, and highlighted the roles and responsibilities of patients as well as healthcare systems and staff [5]. Risks appeared to be especially likely to arise where different agents interact or fail to do so. This reflects findings from a recent study of safe hospital discharge, which determined “threats to safety are located between care providers, processes and settings” [6].

Rhodes et al. described how patient safety in primary care is ‘co-produced’ by patients and staff, with both having the potential to degrade or promote safety [5, 7]. The importance of patient involvement in healthcare and its impact on patient safety is increasingly being recognised [8]. As the only constant actor in their healthcare, patients may be best placed to manage their safety in this setting [8, 9].

One patient group particularly likely to be at risk of patient safety incidents is older people with multimorbidity (defined here as having two or more long-term conditions) [10]. Patients in this population can have many health problems, complex needs, and frequent interactions with healthcare staff in a number of different clinical contexts. This presents a range of challenges for GPs [11, 12]. In addition, older patients are more likely to be frail and, thus, potentially more vulnerable to harm if exposed to a patient safety incident [13–15]. Despite this, little is known about how and when risks to patient safety arise for this group. Authors of the recent review of patient safety incident reports, described above, separately analysed a subsample relating to patients aged 65 or over [16]. More than half of these reports described harm to patients, with the three main sources being medication, timely transfer of information between settings, and clinical decision-making. Medication issues included giving the wrong drugs or dosage, or administering medication at the wrong time. Information transfer within the primary care team or across service boundaries were the most common communication issues. Clinical decision-making problems were the least frequently reported but often related to harm, and included errors in both assessment and treatment decision-making.

New guidelines for the management of patients with multimorbidity suggest the adoption of a ‘tailored’ model of care for some patients, with greater emphasis on shared decision-making to reduce treatment burden and risks associated with healthcare [17]. However, the guidelines say little on how best to involve older, multimorbid patients in their healthcare, and the evidence base is very limited [18].

Older patients with multimorbidity could have the most to gain from increased involvement around patient safety but their capacity for involvement may be constrained by the very characteristics that place them at greater risk. However, some will not be managing their conditions alone, and will have social resources and networks to draw on for support [19]. Thus, it is also important to consider the role of informal carers in patient safety.

Before new patient- and carer-centred interventions to reduce risks to patient safety can be developed, we need a better understanding of how risks arise for older, multimorbid people as they interact with primary care services and manage their conditions, and how they respond when their safety is threatened. To do this, we used a longitudinal, qualitative, case study approach, following a group of older patients with multimorbidity on their healthcare journeys for approximately 2 years, collecting data from multiple sources.

When planning this study, we developed a taxonomy of events that could lead to patient harm by collating data from three previous qualitative studies of patient safety in primary care [20]. This taxonomy contained five domains: access, communication, coordination/management, relationships, and technical matters. In this article, we build on our initial taxonomy, identifying and describing threats to patient safety reported by older people with multimorbidity at the start of the study.

## Methods

MAXimising Involvement in MUltiMorbidity (MAXIMUM) in Primary Care was an ethnographic longitudinal, qualitative study of patient safety. The full protocol is published elsewhere, and a COnsolidated criteria for REporting Qualitative research (COREQ) Checklist has been completed for this manuscript (see Additional file 1) [20]. Our aim in this article is to identify and describe the experiences and perceptions of older people with multimorbidity in relation to known and emergent threats to patient safety.

### Sample and recruitment

Details of the study were circulated to GPs in Greater Manchester, through Clinical Commissioning Groups, Local Medical Committees and direct mailings. Recruited GPs were asked to identify a purposive sample of potential patient participants and mail them invitations to the study. To be eligible for inclusion, patients had to be known to the GP, able to communicate in English, aged 65 or over, and have two or more active, long-term, physical health conditions. GPs were instructed to prioritise those who may be especially vulnerable to patient safety incidents and harm, but exclude people who lived in care homes, were likely to die within the next 12 months, or lacked capacity to provide informed consent. Our protocol and ethical approval included provisions in case individuals lost the capacity during the course of the study [18]. GPs were also asked not to select patients they considered themselves to have a “problematic” relationship with or who already had “an awful lot on their plate” as we were mindful of the potential to over-burden a patient or make a bad situation worse.

Interested patients contacted the research team directly, by telephone or returning a reply slip. A member of the research team then spoke to the patients about the study, answered questions, checked understanding and, where appropriate, arranged to meet to take written, informed consent and carry out the initial interview.

### Data collection

All recruited patients participated in an in-depth, semi-structured interview about their health and healthcare at baseline. The interviews took place between July 2014 and August 2015 in participants’ own homes, and had an average duration of 32 min. The majority of participants were interviewed alone. However, one had a friend present and others had a spouse present for some or all of the interview. Where these individuals wished to contribute, informed consent was sought for their participation.

Interviews were conducted by two of the authors, Rebecca Hays (female, Research Associate, MPhil) and Gavin Daker-White (male, Research Fellow, PhD), who are both experienced qualitative researchers. Prior to each interview, the researcher explained they had no medical training but were interested in patient safety, healthcare experiences, communication and relationships in primary care. Interviews were audio-recorded, and the researchers made field notes; completing a medical information form for each participant during the interview (see Additional file 2) and making further notes immediately afterwards.

Conceptually, our study was informed by a theoretical framework derived from the existing literature on patient safety in primary care, as discussed above. The medical information form was drawn up with two goals in mind: to facilitate comparison of interview data with that collected in other ways during the longitudinal study (e.g. via observations or from health diaries); and to capture participants’ experiences and perceptions of healthcare and the details of their health and wellbeing, medical conditions, prescribed and other treatments, and use of different health and social care services. Whilst participants were aware of the nature of the research, we avoided specifically prompting participants about “patient safety” so as to avoid potential biases in data collection based on any pre-conceived notions of what this entailed.

### Sample and participant demographics

Patients were recruited from 5 general practices in Greater Manchester, with a range of 1 to 9 participating patients per practice. These were located in central and suburban areas, including 2 in the lowest decile of the Index of Multiple Deprivation (IMD), which indicates a high level of deprivation [21]. Participating practices also varied in terms of patient list size, with 1 being particularly large and 1 below average.

After receiving a letter of invitation and an information sheet from their GP, 31 patients contacted the research team. Of these, 2 did not wish to participate and 3 felt it would be too much because of other health-related commitments. Thus, 26 older patients with multimorbidity were recruited (Table 1).

**Table 1:** Demographics of patient participants (from patient reported data at baseline)

		Number	Percent
Gender	Female	15	57.69
	Male	11	42.31
Age group	65–74	10	38.46
	75+	16	61.54
Prescribed medicines	≤6	7	26.92
	7–10	8	30.77
	11+	11	42.31
Long-term conditions	2–3	7	26.92
	4–5	7	26.92
	6+	12	46.15
Requires mobility aid	Yes	9	34.62
	No	17	65.38
Living status	Alone	7	26.92
	With partner	16	61.54
	With family member	3	11.54
Index of Multiple Deprivation (IMD) Decile^a^	1–2	9	34.62
	3–4	12	46.15
	5+	4	15.38
Total number of patients		26	100.00

^a^Where 1 is most deprived 10% of Lower Layer Super Output Areas (data from [33])

At the time of recruitment, patients’ ages ranged from 66 to 87 (mean 76). On average, they reported having 5 long-term conditions (range 2–8) and 10 prescribed medicines (range 3–18). Their conditions included painful and respiratory conditions, hypertension and coronary heart disease, thyroid and prostate disorders, diverticular and chronic kidney disease, diabetes, anxiety, stroke, psoriasis and glaucoma. The count of medicines included items such as emollient creams but not adjuncts such as needles and test strips. Approximately one third of patients lived alone (7, 27%), and approximately one third required a mobility aid (9, 34%). The majority lived in deprived areas (21, 81%), where people are more likely to be multimorbid from a younger age [12].

### Data analysis

Interviews were audio-recorded, transcribed, and anonymised; and managed in NVivo 10 alongside the researchers’ field notes. These documents were analysed thematically using a framework approach to identify threats to patient safety in and around primary care [22].

Initially, all transcripts were read and re-read by one researcher (RH), who coded emerging themes. Themes were organised into a framework based on a taxonomy of events that could lead to patient harm, developed when designing this study [20]. A further two domains, health and treatment, were added to the existing five to encompass all emerging themes before this structure was revised and the final framework agreed. The analysis was also informed by a meta-synthesis of qualitative studies of patient safety in primary care that emphasised the need to consider the roles and responsibilities of all agents in healthcare, including systems, and how these interact or fail to do so [5].

The developing coding framework was shared and discussed with the whole research team. This includes health services researchers with medical sociology and psychological science backgrounds, GPs, and patient and public involvement members; thus, increasing the credibility and trustworthiness of the analysis [23, 24]. Uncertainties about relevance to patient safety were discussed, and codes were revised or removed to better summarise and reflect the threats reported by participants. For example, ‘kidneys damaged due to poor diabetes control’ became ‘preventable harm due to poor condition management’ and finally ‘complication of condition - or concern about’. Particularly important issues, in terms of impact on patient safety and need for intervention, were drawn out and are the focus of this article.

### Patient and public involvement

Interpretations of the findings were not checked with participants. However, the MAXIMUM study has had patient and public involvement since the outset, from authors Wendy Barlow and Brian Minor. They have contributed to study design, development of materials, study management, and data analysis. Concurrent to data collection, they have independently analysed a stratified random sample of transcripts. Emerging themes were discussed and compared to those identified by a researcher (RH). This led to the coding framework being expanded and revised.

## Results

### Threats to patient safety

Patient reported threats to patient safety were organised into six themes, across three domains of health and care (Table 2). We provide examples of each and highlight the issues that could have the greatest impact on patients, and those that may be amenable to patient- and carer-centred intervention. Participants’ names have been changed to protect their identity.

**Table 2:** Threats to patient safety reported by older people with multimorbidity

Themes	Examples
Everyday management of:	
Health	• Struggles to accept limitations and does too much. • Views old age as cause of or reason for health problems.
Treatment	• Has side-effects of medication or treatment. • Limits use of medication or treatment.
Primary care:	
Access	• Finds it difficult to get an appointment, esp. with preferred provider. • Restricts primary care attendance.
Coordination	• Received contradictory or conflicting information or advice. • Unable to obtain prescribed medication from pharmacy.
Breakdowns in:	
Communication	• Did not receive results or feedback. • Is unforthcoming about health problems.
Relationships	• Sees healthcare staff as unhelpful, rude, or disrespectful. • Feels they are not always believed.

(The full coding tree can be found in Additional file 3)

#### Everyday management of health

Patients’ experience of and response to their health had implications for patient safety. Pain was commonly reported by participants, and could have a big impact on their sleep, grip, balance, and mobility. This led patients to seek different ways to manage pain, from potentially contradictory sources:

*“I have gone to see different people … they all tell you different things … you’ll try anything when you’re in pain.”* (Deborah, interview transcript)

Threats to patient safety also arose where patients struggled to accept their functional limitations. Some appeared frustrated by their difficulties and, at times, tried to move too quickly or do too much. On a few occasions, this led to injury as a result of falls when, for example, throwing a football back over a wall or carrying too much shopping. Some patients identified how their behaviour had contributed to these incidents but others blamed cumbersome mobility aids, or questioned the quality of joint replacements.

*“I had a fall … my own fault doing something stupid.”* (Ruth, interview transcript)

Those who had sustained injuries appeared to fear falling again, and tried to avoid certain activities, such as going for walks during Autumn and Winter when the ground may be slippery. However, this could further threaten patient safety by reducing mobility and fitness, which could increase social isolation and effect patients’ ability to manage their weight and health through exercise:

*“…with my bones being creaky, I can’t exercise as much and because I can’t exercise the weight is going up. When the weight goes up that contributes towards cholesterol so it doesn’t help.”* (Richard, interview transcript)

Patients worried about their health declining and whether they were developing additional problems, such as dementia. These concerns persisted when patients did not discuss them with a care provider:

*“Memory has changed, not spoken to anyone, hope it’s not Alzheimer’s.”* (Deborah, field notes)

However, patients often attempted to normalise their symptoms in relation to age. This perspective was substantiated when providers indicated particular health problems were “not uncommon for people of my age”, and by the similar experiences of friends and family:

*“I don’t sleep very well but that’s another one but then again I put that down to old age because everyone I know doesn’t sleep…”* (Ruth, interview transcript)

Issues arose when patients were not aware how serious certain symptoms or conditions could be. One person developed chronic kidney disease before learning more about his diabetes and how to manage his diet.

Although most patients did not report mental health problems, a number talked about stress and low mood in relation to their health, care, or social circumstances. In some cases this led to them feeling overwhelmed by and declining further treatment:

*“I’ve had enough. I’m getting stressed out with it all. I just don’t want another one [operation]. I’m too stressed out. … I can’t do it [put weight on joint] and it gets me. It hurts…”* (Edward, interview transcript)

#### Everyday management of treatment

As expected, patients highlighted many concerns relating to their experience and management of medication. Side effects were of particular concern and had the greatest impact on quality of life. Statins, inhalers, and painkillers were most commonly implicated.

One patient described the “dreadful effect” statins had had on him; an experience made worse when he reported this to his care providers but “wasn’t believed”. He felt as though he had been “put through hell” at the hands of doctors who “get points” for prescribing this type of medication. Another patient described the “vicious circle” that can arise when a side effect of a medication worsens the condition they take medication for:

*“I’ve put on a lot of weight. That’s another problem you see because with the COPD [chronic obstructive pulmonary disease] they tell you not to put on any weight. It’s bad for you but you put it on because of the steroids.”* (Ruth, interview transcript)

A number of patients knew why they were taking medications but struggled to remember or pronounce their names and tended to differentiate according to shape, size, and colour. This could potentially cause confusion when patients needed to discuss medications with care providers, request repeat prescriptions, or if the appearance of a medication changes. At times, these problems appeared to arise because of the sheer volume of healthcare information patients needed to retain:

*“I do get occasional problems where I have to use the, what’s it. I’ve forgotten the name of that as well now, you can see how much of my brain is filled up with these medical things…”* (Michael, interview transcript)

Whilst some patients received blister packs put together by their pharmacist, others relied on a family member to manage their medications. However, carers could become overwhelmed and confused when changes were made:

*“Are they blood pressure? I don’t know, they changed them you see. He’s just had them all changed and I’m not with it myself.”* (Edward’s wife, interview transcript)

Some patients sounded flippant when referring to the sheer quantity of medications they take but also expressed reluctance to take yet more. One patient described medications as “the bane of my life” and some preferred to live with minor health problems rather than take extra tablets. Others seemed concerned about all aspects of their life being medicalised, or the combined effects of the medications they take and their potential to cause harm:

*“I don’t like taking tablets … because it’s not a natural thing to be doing. I don’t think. … It must be doing something wrong to your insides.”* (George, interview transcript)

Patients did admit to making mistakes in taking medications, such as missing a dose, but more frequently mentioned intentional non-adherence, especially in relation to painkillers. Patients, particularly those with diabetes, also talked about being unable or unwilling to follow other medical advice, with some choosing quality of life over strict adherence:

*“…if you live like they want you to live you wouldn’t have a life … you’ve got to enjoy what you’ve got while you’ve got it.”* (George, interview transcript)

#### Primary care access

Patient safety could be threatened by patients’ difficulties accessing care when it was needed, or failing to access care when it was required.

Patients reported difficulties contacting their GP surgeries by telephone and making appointments, especially to see a preferred provider. Getting around these difficulties required active management:

*“…my biggest problem is getting in to see the doctor, because you want to see your own doctor. So I’m usually there at half past seven in a morning, if I get there at half past seven, I can get myself an appointment for the same day.”* (Elizabeth, interview transcript)

Some services seemed to place responsibility for managing appointments on the patient, whereas others sent reminders. Where GP surgeries normally assumed this responsibility, some patients appeared to depend on these reminders, and would wait to be contacted even when they were aware appointments were overdue:

*“…it’s supposed to be happening about now but it hasn’t happened, I don’t know. I don’t set that up. It comes from them or else it won’t happen”.* (Thomas, interview transcript)

The patients’ role in accessing care was further highlighted by those who reported not having seen a dentist for years, and restricting or delaying attendance at their GP surgery, preferring to try and diagnose or deal with health problems themselves. For some, this appeared to be about avoiding the “bother” of an appointment but for others the approachability of care providers was an important factor. Failures to access care could also represent uncertainty about what symptoms were a ‘normal’ part of aging and what is a ‘good enough’ reason to request an appointment:

*“It would be useful to have a booklet, ‘what to expect when you get older’, so you know when to go to the GP.”* (Ruth, field notes)

#### Primary care coordination

Participants described a lack of effective coordination of care, and threats to patient safety were identified when appointments were not aligned to patients’ needs and when they did not receive helpful information from providers.

Patients with diabetes felt their care could be better coordinated, to enable more timely intervention and reduce the likelihood of developing complications. For example, blood glucose tests were carried out months before or immediately after consultations with specialists. Thus, discussions concerned dated results that did not reflect their current health status, and patients did not have the opportunity to seek timely advice about how they could improve their self-management.

Ineffective communication between patients and staff was particularly evident when patients were unsure about who had referred them to another service and why, or when they experienced difficulties obtaining medications. In one instance a patient described how an inhaler she used regularly had been taken off her repeat prescription list and how she’d had to convince a GP that it was necessary:

*“I couldn’t understand why she’d crossed it out. I mean she shouldn’t have done that. … Four puffs four times a day is what it said on the prescription and I said add it up and when she added it up she realised, yes, I did need what I was getting but it took a lot of convincing with her. I was very angry over that.”* (Ruth, interview transcript)

Whilst patients continued to attend review appointments with practice nurses, some expressed concern that these were a waste of time and NHS resources, and questioned the point of being asked “the same questions all over again”. This was especially true when patients did not “feel any wiser” after an appointment, and seemed to be compounded by care providers’ explanations that they have “got to fill all this in”.

A number of patients had received contradictory information. Instances included perceived conflicts between NHS care providers and complementary therapists, different primary care providers, GPs and specialists, or even from the same GP. Patients were left feeling confused about what conditions they had, the nature of their conditions, how these could be managed and how well controlled these were, as well as what medications were needed. These conflicts were experienced as distressing for patients who then had to manage and decide what to do in such situations.

*“…he said you have a 40 per cent chance of a heart problem or a heart attack within the next ten years. Hardly looking at me to tell me this … and your diabetes is poorly controlled. … I came away and I was really, I was quite upset by it and … my wife said ring your consultant … He was angry that I’d been told that my, I was a poorly controlled diabetic. … he said words to effect that that doctor does not know what it means to control diabetes”.* (Thomas, interview transcript)

Contradictions also arose from discrepancies between a person’s experience of health and healthcare and that of their friends or relatives. Such uncertainty could be compounded when patients’ symptoms were normalised in the context of their older age.

*“…how did they come to the conclusion I’d got it because I just happened to go to the doctors one day, feeling a bit out of breath, and she put me on this machine … and that was all I had to diagnose that I’d got COPD. Now, other people I know they’ve been sent to hospital and had all different tests done…”* (Ruth, interview transcript)

Coordination issues also arose in relation to community pharmacies, with patients finding that prescribed medications were not available when needed. One patient suggested such problems arose because of a lack of organisation and planning by the pharmacy and a lack of communication between the pharmacy and general practice. As well as leaving patients without medication, these issues necessitated repeated visits and phone calls by already burdened patients, and appeared to create additional work for both pharmacists and GPs.

#### Breakdowns in communication

Communication issues arose in relation to patients’ health and treatment. In some instances patients reported not being told what was wrong. This seemed particularly evident when patients had been in hospital but the reason for their symptoms was not communicated at the time or since. Patients’ appeared to deal with this uncertainty by developing their own theories about the cause of their health problems or just hoping it would not happen again:

*“Nobody explained what had happened at all in the hospital. All they were doing was making your chest better, which is fine and fair enough, but nobody ever said why I had got a bad chest.”* (Thomas, interview transcript)

Lack of communication also resulted in some patients being unaware of how and when, or when not, to take medications or supplements, until these were discussed with a pharmacist at a later date.

Lack of communication between different healthcare staff produced delays for patients and extra work for all involved. As noted above, patients reported difficulties obtaining repeat prescriptions. At times, this was because their pharmacy had no record of those medications being reviewed by the GP or of blood tests being carried out.

Patients found they had to repeat themselves and request copies of letters when their information was not successfully passed on to their other care providers. One patient who did not do this attended an appointment where neither she nor the care provider understood the reason for referral:

*“He said I don’t know why they’ve send you [Frances], I’ve nothing … he said what’s the matter? I said well, I don’t know, I said because I have everything what you’ve given me, so that were it.”* (Frances, interview transcript)

Patients reported operating on the assumption that ‘no news is good news’ when it came to test results, as these were often not communicated:

*“…I see [practice nurse] for the diabetic checks or blood tests. … I had to come in here for another blood test, but I’ve not heard anything since, so I take it, it’s alright.”* (Elizabeth, interview transcript)

As noted earlier, some questioned the point of their long-term condition review appointments, as these were repetitive with little opportunity to have a discussion and learn from the consultation:

*“‘Don’t feel wiser’ after COPD review with practice nurse, ‘would rather have a discussion’, nurse just checks.”* (Ruth, field notes)

In some cases, the patients themselves appeared unforthcoming about their health problems, seemingly preferring to think of themselves as “lucky” or their conditions as “nothing to worry about”:

*“During the interview, Martha seemed to be breathless, she also had difficulty walking … but she did not report problems in either of these areas.”* (Martha, field notes)

#### Breakdowns in relationships

Breakdowns in communication related to distinct episodes of information sharing but these could have wider implications for patient-provider relationships; and breakdowns in relationships were particularly significant for patients. Effects could be seen on their attitudes toward individual providers, help-seeking behaviours, and mental wellbeing.

Most patients expressed a preference to see the same care provider at each appointment but a lack of relational continuity was not the only reason why relationships suffered. The limited time available within GP consultations left some patients feeling as though appointments were over before they began. In these cases, patients felt “awkward” about approaching their GP, especially in relation to lifestyle concerns such as diet.

*“…when I do [see the GP] it’s a case of in and out … they show you the door as soon as you get in … I’d like to not feel the way I feel when I go to the doctors because believe you me I feel as though I no rights to be going. You don’t get a warm welcome let’s put it that way.”* (Ruth, interview transcript)

Some patients were concerned about how knowledgeable their primary care providers were and expressed a preference for seeing specialists in relation to their long-term conditions. Although the perceived competence and expertise of staff had an impact on patients’ perceptions of their care, the manner of staff was particularly important. Some providers were seen as “better” because they were more likeable:

*“…the nurse I saw last time, she was very nice… some are better than others and, I’m not saying better but you know, she, well, I liked her very much…”* (Gloria, interview transcript)

Relationships had broken down where healthcare staff, including receptionists, were perceived as unhelpful or rude. Examples included care providers eating during, or looking at their computer screens throughout appointments; not asking if it was okay to have students present; and criticising patients for their actions or help-seeking behaviours rather than offering assistance or advice:

*“It’s as though [GP] doesn’t want to bloody touch you. A normal gentleman would help you on with your coat because I was really struggling and it was, he didn’t want to do it because he said to me you should have put the other arm in first.”* (Ruth, interview transcript)

Patients seemed annoyed, angry, and offended when discussing these encounters but it was their experiences of not being believed that seemed to leave them feeling most vulnerable. Difficult situations became more troublesome when healthcare staff were seen as portraying themselves as infallible and their patients as wrong:

*“With some doctors … the ones that think they’re absolutely right, they know it, the statistics tell them that and the computer tells them that, they are the ones that are really difficult to deal with.”* (Thomas, interview transcript)

One patient reported great success at managing a number of his health problems through diet, in combination with medications where necessary. He found it impossible to work with providers who did not take this seriously, and vowed “never to go to” those doctors again.

The sense of being misunderstood seemed to threaten a patient’s identity, and led to some taking steps to prove what they were saying to their care providers, such as taking themselves off then re-starting a medication to confirm whether the symptoms they were experiencing were indeed side-effects [25].

In some circumstances, patients had to be forcefully or quietly persistent to obtain much needed medication before they ran out of supplies; or a diagnosis that made sense to them, helped them manage their health, and improved their wellbeing.

*“That fibromyalgia is really debilitating … I could hardly walk about until it was diagnosed. They just kept saying it was arthritis but I was so weary. Then I went to see another doctor … and he said straight away what was the problem, and actually knowing you’ve got a problem it takes a lot of the stress away when people say they don’t believe you … once you know that you’ve got something you face up to it and you can tackle it better.”* (Brenda, interview transcript)

## Discussion

These baseline findings from a longitudinal qualitative study highlight how and when patient safety can be threatened in primary care. We found threats to patient safety were intrinsically linked to the challenges people face when managing health conditions, navigating the healthcare system, and negotiating care. This includes a number of issues already highlighted in the literature, for example, on medication adherence and accessing primary care [26, 27]. However, we have collated and expanded the existing evidence base by identifying and bringing together the risks most pertinent to older people with multimorbidity, and establishing the extent to which they can influence their own patient safety.

A number of threats to safety appeared magnified for older patients with multimorbidity as they attempted to: make sense of their health in the context of multiple long-term conditions and older age; manage multiple medications and seek a balance between their health and quality of life; and know when and how best to access care, as well as how to cope with the way services are coordinated. Our research has highlighted a lack of communication around these issues, and how it is bounded by the status of patient-provider relationships.

For patients, their experience of healthcare appeared to have the biggest impact on safety in primary care as perceptions about the quality and quantity of interactions with staff and services influenced their subsequent behaviour. Thus, as previously reported, patient safety can usefully be thought of as a continually renegotiated and co-produced feeling [5, 7].

Where patients’ needs and concerns go unaddressed, and particularly where their personal identity is threatened, patients can be left feeling unsafe [25]. Such feelings of unsafety can remain hidden but have important consequences, affecting relationships with healthcare staff, and patients’ self-care and consulting behaviour.

Our findings can be contrasted with recent studies using reviews of incidents [16]. There were significant areas of overlap, including the importance of medication and communication issues, but the incident reviews were more likely to identify clinical decision-making, and information transfer *between* teams, neither of which are likely to be transparent to patients. The priority afforded to communication in the present study aligns with a recent Delphi exercise on the burden of iatrogenic harm in primary care [2].

Our findings also add to the growing understanding of the important role of patients’ in patient safety. The threats that appear most amenable to patient-centred interventions concern patients’ experience and everyday management of their health and treatments, and their access to and communication with primary care services and staff. The coordination and organisation of care was the one area where individual patients appeared to have little or no influence, and were only able to respond to failures as they arose.

Our findings identified ‘everyday management of health’ as a theme. There is an argument that such issues are outside the formal remit of patient safety, as that is usually defined as negative outcomes “…stemming from the process of healthcare” [1]. We have included this theme here because we believe it is highly relevant to patient safety in the context of multimorbidity as everyday management can have important consequences for later patient safety incidents. Self-management is considered critical for the prevention and management of long-term conditions, and any consideration of self-management needs to consider the social context in which patients live and the influence of self-management support on the lives that patients live outside of healthcare settings [28–31]. Thus, a strict distinction between healthcare and everyday settings may be less relevant in the current study, although we accept it is important to be aware of the issue.

### Implications

This research has important implications for the development of interventions to improve patient safety. It enables us to identify potential mechanisms for involving older patients in their healthcare and patient safety as recommended in new guidelines for the management of multimorbidity [17]. The knowledge of the dynamic nature of patient safety, which this study adds, could be exploited to positive effect. As patients’ perceptions influence subsequent behaviour, interventions that affect their experience and interpretation of healthcare interactions have the potential to influence patient safety outcomes.

Whilst patients do not necessarily have a good understanding of what safety means in the context of healthcare, our results reflect the findings of previous qualitative research in this area that shows patients can report relevant issues when questioned [7]. In these baseline interviews, patients were able to describe the challenges of managing their health and healthcare in the context of their quality of life, and the threats to safety that arose as their journey as a patient unfolded. However, older patients with multimorbidity might benefit from a greater awareness of when and how harms can occur, and what could help keep them safe as this could help them make more informed choices, for example, about when to access services.

Interventions could also aim to provide patients with more realistic expectations for primary care, highlighting that healthcare services and staff are fallible, and the potential for patients to improve their own and others safety in this setting by identifying and speaking up about problems. Thus, another potential mechanism for reducing threats to patient safety involves supporting and empowering patients to communicate their needs and concerns more effectively. Such an intervention could open pathways to more efficient and empathic communication between patients and care providers, and reduce risks to patient safety. In doing so, it would need to overcome the tendency of older, multimorbid patients to downplay their problems. The existing evidence base for this type of intervention is very limited but promising [18].

A fundamental question to be addressed in the development of patient-centred interventions to improve patient safety, is how can patients be helped to know when it is important to speak up and be assertive enough to change the course of their care without engendering defensiveness in healthcare staff or further breakdowns in the patient-provider relationship [27].

We have focused on identifying ways in which patients could be supported to increase their involvement in healthcare, but our findings also have implications for healthcare services. Such interventions could prioritise those areas that fall outside of patients’ influence, for example, the coordination of care within and between services.

By reviewing what happens over time and incorporating the perspectives of formal and informal care providers, our ongoing research through the MAXIMUM study will further explore how threats to patient safety arise in primary care, what situations result in patient safety failures, and how risks can be mitigated and failures avoided. We will also be mindful of and explore the feasibility of potential patient-centred interventions throughout. In addition, further patient and public involvement will be carried out to determine what this vulnerable, older, multimorbid population has the capacity to do, and how such individuals can be engaged in patient safety vigilance and management.

### Strengths and limitations

Our study adds to the developing evidence base concerning patient safety in primary care [2, 5]. Our prospective study is designed to explore how incidents arise in older patients with multimorbidity, and how patients and professionals respond. These methods complement previous studies which have explored reported incidents to assess possible causes [2, 4]. The latter provides a rich set of data for generation of hypotheses but our prospective methods may be more suited to identifying those factors that lead to patient safety issues arising.

Despite our multifaceted approach, we experienced difficulties recruiting GPs to the study. Some may have been reluctant to participate in a study of patient safety. However, the main difficulty appeared to be the capacity of practices to participate in research. This was also evident among the recruited GPs, some of whom struggled to identify and contact potential patient participants, even after we simplified our recruitment criteria by making it a request rather than a requirement to prioritise those who may be especially vulnerable to patient safety failures.

Feedback from the GPs indicated they had limited capacity because of the various demands on their time, which were often accompanied by staff shortages and changes within their practice. Thus, our experience of attempting to recruit general practices to participate in the MAXIMUM study reflected those of the patients in trying to access care. It also highlights a potential cause of patient safety incidents in primary care, and reinforces the Royal College of General Practitioners call for action to be taken at local and national levels to address barriers to the provision of safe and effective care for patients with multimorbidity [32].

Whilst we did not experience such difficulties recruiting patients, we do not know exactly how many people were sent letters of invitation to the study by their practices. Factors such as the length and intensity of the study are likely to have affected recruitment. Thus, different results may have been obtained through a one-off interview study.

We did not achieve data saturation but did not set out to as the data analysed were collected at baseline of a longitudinal study. Using an *a priori* approach to analysis allowed us to focus on and expand understanding of recognised issues of importance in primary care patient safety. We were also able to identify additional areas of concern and assess their importance and potential impact. However, the relationship between threats to patient safety and patient safety incidents needs to be explored further, to identify the threats and circumstances that lead to adverse outcomes or injuries, and whether or not it is possible to avoid, prevent or ameliorate the risks they pose.

## Conclusions

For older patients with multimorbidity, patient safety is intrinsically linked to the challenges people face when managing health conditions, navigating the healthcare system, and negotiating care. We have considered and will continue to explore the implications of this for the development of patient-centred interventions to reduce risks to patient safety. The findings from the MAXIMUM study, including this analysis, will then be used to develop and test ways of raising awareness of potential threats, developing more realistic expectations for primary care, and affording patients with more opportunities to speak up when they have concerns for safety.

## Additional files

Additional file 1: COnsolidated criteria for REporting Qualitative research (COREQ) Checklist (DOCX 27 kb)

Additional file 2: MAXimising Involvement in MUltiMorbidity (MAXIMUM) in Primary Care - Patient participant medical information form (DOCX 50 kb)

Additional file 3: Data coding tree (DOCX 22 kb)

## Abbreviations

COPD: Chronic Obstructive Pulmonary Disease
GP: General Practitioner
IMD: Index of Multiple Deprivation
MAXIMUM: MAXimising Involvement in MUltiMorbidity in primary care
