# Supplementary material for: Threats to patient safety in primary care reported by older people with multimorbidity: baseline findings from a longitudinal qualitative study and implications for intervention
Source: BMC Health Serv Res. 2017 Nov 21;17:754. doi: 10.1186/s12913-017-2727-9 (PMC5697352; doi:10.1186/s12913-017-2727-9)
Supplement: Supplementary file 2 — MAXimising Involvement in MUltiMorbidity (MAXIMUM) in Primary Care - Patient participant medical information form (DOCX 50 kb) [file 12913_2017_2727_MOESM2_ESM.docx]

**MAXimising Involvement in MUltiMorbidity (MAXIMUM) in Primary Care**

**Patient participant medical information form**

| **Patient participant ID number:** |  | | |
| --- | --- | --- | --- |
|  |  |  |  |
| **Date form completed:** |  | | |
|  |  |  |  |
| **Time period (select one):** | 0 months | 12 months | 24 months |

*The following information should reflect the current health status of the patient participant referred to above:*

| **Main symptoms:** | **Medical conditions and health problems:** |
| --- | --- |
| **Current medications:** | **Known allergies:** |
|  | **Other current treatments:** |
| **Other professionals involved in this patient’s care:** | **Active referrals (including clinics within the General Practice where relevant):** |
| **Details of scheduled appointments:** | |
