# Supplementary material for: Threats to patient safety in primary care reported by older people with multimorbidity: baseline findings from a longitudinal qualitative study and implications for intervention
Source: BMC Health Serv Res. 2017 Nov 21;17:754. doi: 10.1186/s12913-017-2727-9 (PMC5697352; doi:10.1186/s12913-017-2727-9)
Supplement: Supplementary file 3 — Data coding tree (DOCX 22 kb) [file 12913_2017_2727_MOESM3_ESM.docx]

**Data coding tree**

| **Themes** | **Codes** |
| --- | --- |
| Everyday management of health | Complication of condition - or concern about |
|  | Condition or symptom of most concern to P |
|  | Fear related to health |
|  | Mental health problems including stress |
|  | Mobility problems |
|  | P also a carer |
|  | P believes a condition or symptom cannot be improved |
|  | P concerned about developing new condition |
|  | P concerned about weight and impact on health |
|  | P does not know or cannot remember diagnosis |
|  | P experiences hypoglycaemia |
|  | P has fallen or is at risk of falls |
|  | P is in pain |
|  | P is lonely or isolated |
|  | P is prone to infections |
|  | P not noticed or realised seriousness of health problem |
|  | P refers to their potential life expectancy |
|  | P struggles to accept limitations and does too much |
|  | P views old age as cause of or reason for health problems |
|  | Relative concerned about P |
| Everyday management of treatment | Advice difficult or not possible to follow |
|  | C takes responsibility for managing P's healthcare |
|  | Error in medication taking |
|  | Medication or treatment not working (well) |
|  | P cannot pronounce medication when asking for prescription |
|  | P cautious of life becoming medicalised |
|  | P does not follow advice |
|  | P does not know or cannot remember name of medication or treatment |
|  | P does not know or cannot remember what medication is for |
|  | P does not want or is reluctant to take medication |
|  | P forgot or forgets to take medication |
|  | P limits use of medication or treatment |
|  | P refers to the quantity of medications they take |
|  | P takes health advice from friends and relatives or the TV and internet |
|  | Side-effects of medication |
|  | Side-effects of treatment |
| Primary care access | Appointment not face-to-face - no examination |
|  | Delay in or lack of referral |
|  | Delayed or missed diagnosis |
|  | P does not want further treatment |
|  | P does not want test or investigation |
|  | P finds it difficult to contact practice by telephone |
|  | P finds it difficult to get an appointment (with preferred HCP) |
|  | P not accessing care - not appropriate for needs |
|  | P not offered or sent for test or investigation |
|  | P not or not being sent appointments by HCP |
|  | P not registered with dentist or optician |
|  | P not sure when to access care |
|  | P restricts or restricting attendance |
|  | P waits or waiting for HCP to contact them |
|  | Untimely attendance |
| Primary care coordination | Care not joined up |
|  | Contradictory or conflicting information or advice |
|  | Lack of or perceived lack of hygiene |
|  | Medication keeps changing |
|  | Medication not available from or provided by pharmacy |
|  | Medication removed from repeat prescription list or not prescribed |
|  | P allergic to medication or product used in tests or investigations |
|  | P cannot have medication or treatment due to other condition |
|  | P cannot have medication or treatment due to other medication |
|  | P cannot have test or investigation due to other treatment |
|  | P does not know or cannot remember information about a referral |
|  | P does not know why they need to take a medication |
|  | P doubts diagnosis |
|  | P experienced problem when seen by a trainee HCP |
|  | P experienced problem due to HCP following guidelines |
|  | P not gaining knowledge from appointments |
|  | Prescribing error or near-miss |
|  | Treatment not provided according to guidelines |
|  | Waste of time and NHS resources |
|  | Diagnosis or reason for health problem not known by P |
|  | Lack of communication between HCPs |
|  | P asked same questions at each appointment - not a discussion |
|  | P did not understand letter from HCP |
|  | P learned new information about medication from pharmacist |
|  | P not contacted about review or follow-up appointment |
|  | P not disclosed or played-down health problems |
|  | P not given results or feedback |
|  | P received inappropriate letter from HCP |
|  | HCP seen as unhelpful rude or disrespectful |
|  | HCP thinks they are right - P disagrees |
|  | Lack of relational continuity |
|  | P distrusts specific care service |
|  | P feels dismissed or mistreated by HCP |
|  | P has inadequate time with HCP |
|  | P not believed by HCP |
|  | P would prefer to see a specialist |
|  | Some HCPs seen as better than others |
